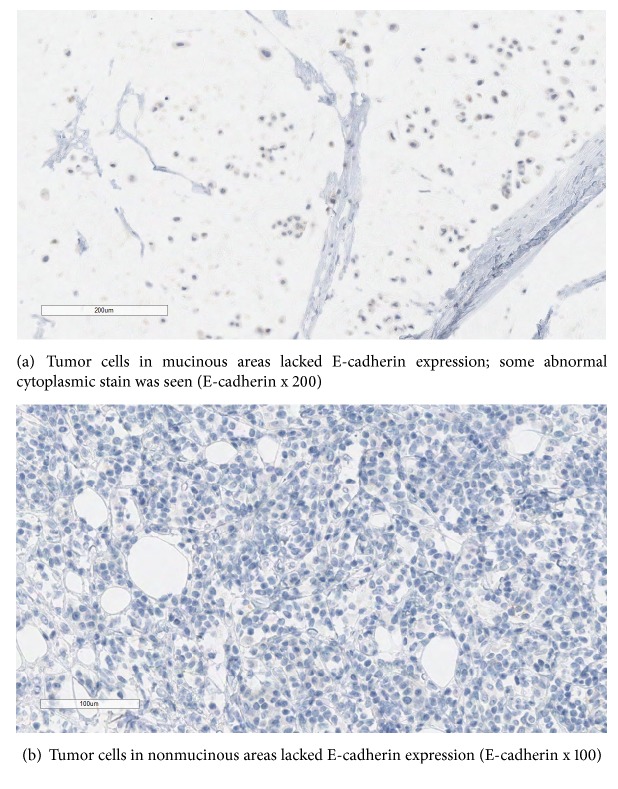# Invasive Pleomorphic-Type Lobular Carcinoma of the Breast Presenting as a Mucinous Carcinoma

**DOI:** 10.1155/2019/1839208

**Published:** 2019-08-04

**Authors:** A. Baig, G. Omeroglu-Altinel, A. Omeroglu

**Affiliations:** Department of Pathology, McGill University, Canada

## Abstract

Invasive mucinous carcinoma of the breast is mostly associated with invasive carcinoma of no special type (NST) and sometimes with neuroendocrine type tumors such as solid papillary carcinoma. Extracellular mucin production in invasive lobular carcinoma (ILC) is extremely rare. To the best of our knowledge only 18 such cases have been described in the literature. Here we present a unique case of invasive pleomorphic-type lobular carcinoma (IPTLC) presenting as a mucinous carcinoma of the breast on core needle biopsy. Here we discuss the impact and ways to suspect such case.

## 1. Introduction

Invasive mucinous carcinoma of the breast is characterized by clusters of neoplastic cells floating in extracellular mucin. Most of the patients are elderly and tumors are generally considered to have a relatively good prognosis when identified as “pure” mucinous tumors. Mucinous carcinoma can be divided into two subtypes: a less cellular, nonendocrine (type A), and a cellular variant with frequent neuroendocrine features (type B). A mucinous tumor is generally accepted as “pure” when at least 90% of the tumor has extracellular mucin. Mixed subtypes usually contain various amounts of invasive ductal NST or tumors with neuroendocrine differentiation. Mixed mucinous carcinomas have a worse prognosis with increased incidence of lymph node metastasis than do pure variants. Invasive lobular carcinoma, which makes 5-15% of breast cancers can show intracellular mucin production, however is exceedingly uncommon to be associated with extracellular mucin production. The pleomorphic subtype is rare comprising approximately 15% of all ILC of the breast and is generally accepted as having a more aggressive behaviour compared to nonpleomorphic variants. It is also associated with relatively increased likelihood of Her2 positivity and lower estrogen and progesterone receptor expression when compared with the classical ILC [1, 2]. Based on our literature review this is only the second case of IPTLC with extracellular mucin production and a unique case with initial diagnosis of invasive mucinous carcinoma on biopsy.

## 2. Case Presentation

**Figure 1:**
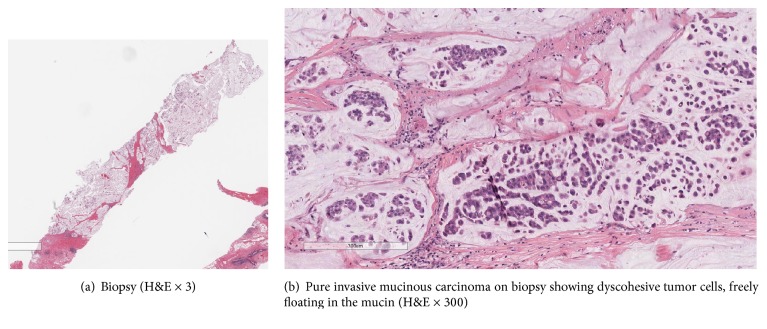


A 67-year-old postmenopausal female with no past medical or family history of cancer presented with a palpable breast mass on examination with no axillary lymphadenopathy. The mammography showed a lobulated 4.7 cm mass in the left breast upper inner quadrant. Biopsy showed a pure invasive mucinous carcinoma (Figures 1(a) and 1(b)). Tumor was ER positive in 5% of cells, PR was negative, and Her2 was equivocal (score 2+) but FISH negative. Due to weak ER expression, the receptor studies were repeated but results did not change. Patient underwent a left breast partial mastectomy and left axillary sentinel lymph node procedure.

### 2.1. Final Pathology

**Figure 2:**
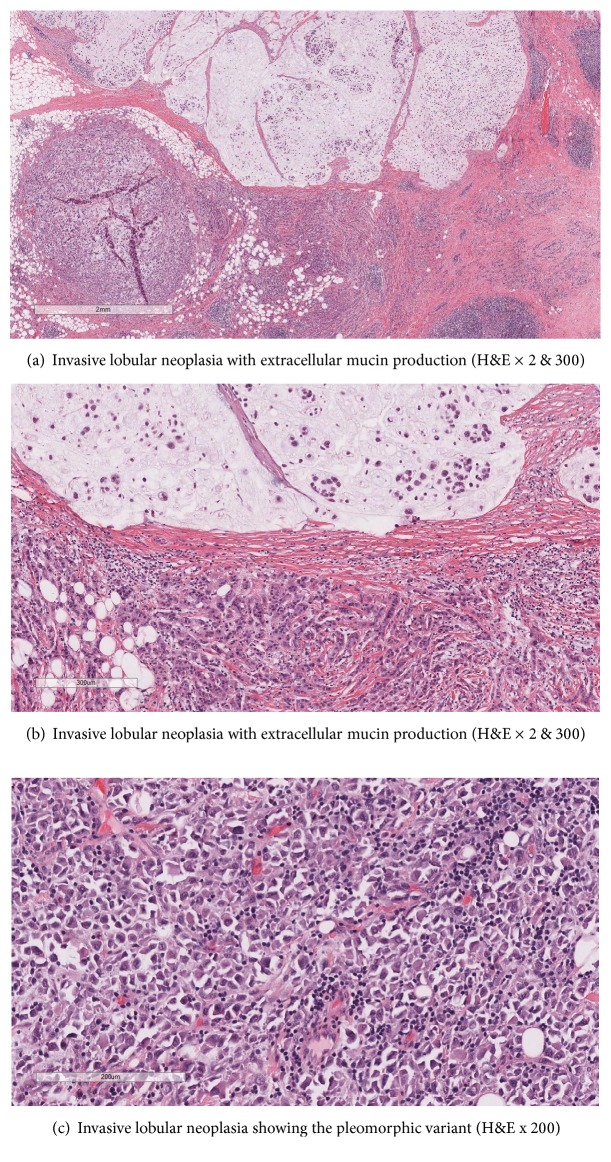


Sections showed a 6.0 cm tumor that volume wise was 50% mucinous (Figures 2(a) and 2(b)). The Nottingham grade in nonmucinous and mucinous component was Grade III (3/3/2) and Grade II (1/3/2), respectively with an overall Grade III out of III. Tumor cells in both mucinous and nonmucinous areas lacked E-cadherin expression (Figures 3(a) and 3(b)) consistent with a lobular phenotype. Histological features of ILCEM have been previously reviewed in literature [3]. In this case the ILCEM is diagnosed as the pleomorphic subtype showing cytologic cellular features with abundant eosinophilic cytoplasm, signet ring, and plasmacytoid cells (Figure 2(c)). Lobular carcinoma in situ (LCIS), also pleomorphic type, was also identified, admixed with invasive carcinoma forming <25% of the mass. The nonmucinous component was ER positive in 60% of cells, PR negative, and Her2 equivocal (score 2+) but FISH negative. The mucinous component differed in that it was ER positive in only 10% of cells; PR and Her2 results were similar. Isolated tumor cells (ITCs) identified on keratin stained section were present in one out of two axillary sentinel lymph nodes.

### 2.2. Follow-Up

The case was discussed in tumor board and the decision was to proceed with adjuvant chemotherapy followed by radiotherapy and hormonal treatment.

## 3. Discussion

In common practice, a diagnosis of mucinous carcinoma or ductal carcinoma with mucinous features is often made in the presence of extracellular mucin, without immunohistochemical confirmation of the ductal phenotype. Intracellular mucin and signet ring cells are common features in ILC but extracellular mucin production is not. A literature review showed eighteen cases of ILC with extracellular mucin production (ILCEM) [3–10]; however a recent publication mentioned that there are twenty-seven such reported cases [10]. Out of all those cases there is only one other possible case of pleomorphic subtype of ILC [3]. To the best of our knowledge our case is possibly the only reported case of a pleomorphic ILC presenting as a mucinous carcinoma.

Predicting that a mucinous tumor might have a lobular origin on the biopsy material requires careful histological/morphological assessment. E-cadherin stain should be performed only in cases that have either a nonmucinous component or a mucinous component with morphology suggestive of invasive lobular carcinoma. In the case presented by Yu et al. 10% of cases of pure mucinous carcinoma studied on tissue microarray showed reduced E-cadherin expression. Positive staining for E-cadherin should not preclude a diagnosis of lobular in favor of ductal carcinoma. Molecular evidence suggests that even when E-cadherin is expressed, the cadherin-catenin complex may be nonfunctional. Misclassification of tumors may lead to mismanagement of patients in clinical practice [11]. Most mucinous carcinomas of the breast show strong ER/PR expression. In retrospect, low ER/PR expression, as in the current case, might have been a clue that mucinous carcinoma originated from a pleomorphic lobular carcinoma. The diagnosis could have been confirmed/facilitated by performing an E-cadherin stain on the biopsy tissue. Applying E-cadherin stain without taking into account the morphology could potentially lead to incorrect diagnosis.

ILC can be frequently missed during screening with mammography due to its diffusely infiltrative pattern and low or equal density compared to normal breast tissue on mammogram. Furthermore, both mammography and ultrasound tend to underestimate the size of the tumor for invasive lobular carcinomas and thus increase the failure rate of breast conserving therapy. Magnetic resonance imaging (MRI) has been proposed as the imaging modality of choice for the evaluation of ILC [12]. As MRI is not routinely performed for nonlobular tumors, suggesting the possibility of an associated ILC on the biopsy material may be relevant to patient's management.

## 4. Summary and Conclusions

Mucinous carcinomas of the breast are usually of either ductal or neuroendocrine phenotype. A lobular origin is extremely rare; however, it is important to identify the lobular phenotype at the biopsy stage due to its impact on patient management. Weak ER expression in an otherwise pure mucinous carcinoma should prompt one to perform an E-cadherin stain to rule out a pleomorphic lobular carcinoma.

## Figures and Tables

**Figure 3:**